# Electroacupuncture Reduces Oocyte Number and Maintains Vascular Barrier Against Ovarian Hyperstimulation Syndrome by Regulating CD200

**DOI:** 10.3389/fcell.2021.648578

**Published:** 2021-02-22

**Authors:** Li Chen, Xuan Huang, Li Wang, Cencen Wang, Xu Tang, Minghui Gu, Jun Jing, Rujun Ma, Xie Ge, Bing Yao

**Affiliations:** ^1^Center of Reproductive Medicine, Affiliated Jinling Hospital, School of Medicine, Nanjing University, Jiangsu, China; ^2^Reproductive Medical Center, The Second Affiliated Hospital of Shandong University of Traditional Chinese Medicine, Shandong, China; ^3^State Key Laboratory of Reproductive Medicine, Nanjing Medical University, Jiangsu, China

**Keywords:** CD200, anti-inflammation, vascular barrier, electroacupuncture, OHSS

## Abstract

Ovarian hyperstimulation syndrome (OHSS) is a common complication caused by ovulatory stimulation therapy, which manifests as an increase in ovarian volume, an increase in the number of oocytes retrieved, and increased vascular permeability throughout the body and especially in ovarian tissue. In our previous study, we found that electroacupuncture (EA) could prevent the progression of OHSS, by mainly affecting ovary. However, the specific molecules and the mechanism of this process were still unknown. In order to explore the underlying mechanism, OHSS rat model was established and EA treatment was performed, which was followed by proteomic analysis of ovaries. Results showed a significant increase in the expression level of CD200 in the ovaries of OHSS group treated with EA than those of OHSS group. Clinical data showed that the level of CD200 in follicular fluid was negatively correlated with the number of oocytes retrieved and serum E2 level. Further *in vitro* experiments showed a concentration-dependent role of human chorionic gonadotropin (hCG) in reducing CD200 and CD200R levels, and increasing inflammatory cytokine levels in cultured KGN cells. In human umbilical vein endothelial cells (HUVECs), the vascular barrier function was improved by CM (cultural medium from KGN cell) which treated with CD200Fc (CD200R agonist). Meanwhile, the results of *in vivo* experiments indicated that EA reduced the number of ovarian corpora lutea, decreased inflammatory response, and improved the vascular barrier function by increasing the expression of CD200 and CD200R in rat ovaries. These findings suggest that EA treatment may reduce oocyte number and maintain vascular barrier against OHSS through ovarian anti-inflammatory response mediated by CD200. Therefore, this study is the first to identify CD200 as a main of EA in the ovary and elucidate the possible mechanism of EA on preventing and treating OHSS, which provide a scientific basis for CD200 as an effector and indicator in EA treatment.

## Introduction

Ovarian hyperstimulation syndrome (OHSS) is a common complication caused by ovulatory stimulation therapy. It is characterized by enlarged ovary, higher number of oocytes, and increased vascular permeability, resulting in redistribution of intravascular fluid to the third space of the body and leading to clinical syndrome which includes ascites, pleural effusion, and oliguria (Veisi et al., 2013; Kwik and Maxwell, 2016). OHSS is attributable to the massive increase in systemic inflammatory cytokines and vasoactive factors present in the serum, follicular, and ascetic fluid, including vascular endothelial growth factor (VEGF), interlukin-1β, IL-6, TNFα, and so on (Loret de Mola et al., 1996, 1997; Pellicer et al., 1999; Artini et al., 2002; Orvieto, 2004). Systemic inflammatory response during OHSS is apparently the consequence of the presence of human chorionic gonadotropin (hCG), which triggers ovulation, enables an increase in oocyte number, and initiates inflammatory secretion in the ovary and human granulosa cells (Orvieto, 2004; Orvieto et al., 2015). Accumulating evidences have supported that activation of NF-κB induces the release of pro-inflammatory cytokines, causing vascular endothelial barrier (VEB) dysfunction (Donato et al., 2008; Xu et al., 2016). Despite plenty of researches have been done, the etiopathogenesis of OHSS has still not been fully elucidated. So, there are no universal guidelines or strategies for OHSS management due to controversies in treatment options.

As an alternative way to medical therapy in treating diseases, electroacupuncture (EA) has been approved and applied by many populations in many countries because it is considered a safe procedure with few contraindications and can be easily applied (Ulett et al., 1998; Kim et al., 2013; Mayor, 2013). A growing number of reports have recently indicated that EA may be effective in treating many types of diseases by regulating inflammatory responses (Kim et al., 2007; Liu et al., 2016; Park and Namgung, 2018). Some studies have reported that EA mainly regulated the NF-kB signaling pathway to ameliorate inflammatory factor (Park and Namgung, 2018). There are also many studies reporting that EA, when it has been applied in artificial reproduction technology (ART), can not only improve the pregnancy rate but also reduce the incidence of OHSS (Cui et al., 2011; Jo and Lee, 2017). However, it has not been clearly identified whether this effect is associated with inflammation suppression.

In our previous study, EA has efficiently rescued the characteristics of OHSS in a rat model, by reducing the size of the ovary and decreasing the levels of sex hormones (Chen et al., 2016). The reduced number of ovarian corpora lutea (CL), promoted luteal regression, and inhibited ovarian steroidogenesis pathway were mainly benefits of EA treatment (Huang et al., 2018). Although studies above have identified ovary as the main target organ of EA treatment, the specific molecules and the underlying mechanism are still unknown. Therefore, in the present study, OHSS rat model was established and EA treatment was performed which was followed by the proteomic analysis of ovaries to identify candidate proteins. The purpose of this study was to further elucidate the effective targets and molecular mechanisms of EA and to provide experimental evidence for the prevention and treatment of OHSS.

## Materials and Methods

### Animals and Study Design

Female Sprague–Dawley rats of 22 days old (D22) were randomly divided into three groups with 12 each. The rats in the control group received 10 IU pregnant mare serum gonadotropin (PMSG) (Ningbo, China) on D27, followed by injection of 10 IU hCG (Ningbo, China) on D29 to induce ovulation; the OHSS rats were administered with PMSG (50 IU/day) for 4 days from D25 to D28 followed by injection of 150 IU hCG on D29; the EA + OHSS (EAO) rats underwent the same hormonal stimulation protocol as OHSS group in addition to EA stimulation for 15 min/day from D22 to D31. All the rats were free to have food and water and maintained at room temperature (21–23°C) with a 12L:12D cycle.

The EA stimulation protocol was performed as described previously (Chen et al., 2016; Huang et al., 2018). The rats in the EAO group were anesthetized with 4% chloralic hydras (0.1 ml/kg) followed by acupuncture at Sanyinjiao (SP6) and Guanyuan (CV4) points with an EA stimulator instrument (Model KDZ-I; Yangzhou Kaida Medical Equipment Co., Ltd., Yangzhou, China). The stimulus parameters included were as follows: 2/15 Hz, disperse-dense wave, 15 min/day. The rats in control and OHSS groups were anesthetized simultaneously to exclude the effects of anesthesia.

The ovaries were collected 48 h later after hCG injection. The ovaries were removed and cleaned of adhering tissue, and four pairs from EAO and OHSS groups were randomly selected for proteomic analysis. The ovaries from other rats were collected for subsequent assays, in which the left ovaries were stored at −80°C and the right ovaries were fixed in 4% paraformaldehyde.

### Proteomic Processing and Bioinformatics Analysis

The ovarian proteins from four biological replicates in each group were extracted using the phenolic extraction method. iTRAQ labeling and quantitative LC-MS proteomics were performed according to the manufacturer’s instructions as described (Piehowski et al., 2013). The mass spectrometer was used to identify the labeled samples from EAO and OHSS group. The quantitative protein ratios were weighted and normalized by the median ratio in Mascot^1^. When the differences in protein expression between EAO and OHSS groups were >1.2-fold or <−1.2-fold (with *P* < 0.05), the protein was considered to be differentially expressed. Gene Ontology (GO) is a standardized gene function classification system that describes the properties of proteins. GO results were included three categories: cellular component, biological process, and molecular function. Based on the proteomic data, KEGG pathway was analyzed to find the key important molecules. The mass spectrometry proteomics data have been deposited to the ProteomeXchange Consortium^2^ via the iProX partner repository with the dataset identifier PXD023493^3^.

### Collection of Follicular Fluid and Serum

Forty-three women aged 21–45 years, BMI between 18 and 25, who underwent conventional ovarian stimulation protocols were studied. During oocyte retrieval under *in vitro* fertilization embryo transfer program, the discarded follicular fluid (FF) samples from the follicles that are larger than 16 mm in diameter were collected. The corresponding serum samples from these patients were retrieved after detecting the hormones, and then were stored at −80°C.

### ELISA for Assessing CD200

The level of CD200 in animal serum, and human serum and follicular fluid were determined using ELISA kit according to the manufacturer’s protocol (SEA880Hu, USCN). The detection sensitivity range of the kits was 4.6 pg/ml and showed no cross reactivity with a series of soluble molecules with a good reproducibility. The coefficient of variation was <12%.

### Cell Culture

The KGN cells were cultured in Dulbecco’s modified Eagle’s medium/Ham’s nutrient mixture F12 (DMEM/F12; Yuanye, Shanghai, China) medium supplemented with 10% fetal bovine serum (Gibco, Thermo Fisher Scientific, United States) and penicillin (100 U/ml) and streptomycin (100 μg/ml) in a humidified incubator containing 5% CO_2_ at 37°C. KGN cells were then seeded into six-well plates (3 × 10^5^ cells/well) and grew overnight till approximately 70% confluence was reached and stimulated with different concentrations (1, 10, and 100 IU/ml) of hCG for 24 h. The untreated medium was used as a negative control in this study. When to investigate the anti-inflammatory effect of CD200Fc in KGN, CD200Fc (100 ng/ml) was pre-incubated for 1 h prior to hCG (10 IU) treatment. After 24 h of incubation, the culture medium was collected and centrifuged at 5000 r/min under aseptic conditions. The suspension was stored at −20°C and served as conditional cultural medium (CM) for human umbilical vein endothelial cells (HUVECs). The culture was then seeded into six-well plates (2.5 × 10^5^ cells/well) and grew overnight till they approximately reach to 70% confluence and stimulated with CM for 24 h. The whole-cell extracts were obtained for biochemical analyses or fixed with 4% paraformaldehyde for F-actin staining.

### F-Actin Stain and Fluorescence Microscopy

After culturing for 24 h with CM, HUVECs were fixed with 4% paraformaldehyde. The cells were then washed with PBS and permeabilized with 0.1% Triton X-100. TRITC-phalloidin (R415, Thermo Fisher Scientific Tech, United States) was diluted 20 mg/ml. The cells were added to the culture in the dark for 30 min. The images were viewed under a fluorescence microscope (IX73, Olympus Corporation, Shinjuku, Tokyo, Japan).

### Western Blotting

The ovary and cell lysates were purified and quantified using BCA assay; 50 μg protein of ovary and 30 μg protein of cells were applied to SDS-polyacrylamide gel and the separated proteins were then transferred onto PDVF membranes. The target proteins were immunoblotted with various primary antibodies after blocking with 5% bovine serum albumin, and corresponding secondary antibodies were incubated by the visualized on Tanon-5200 (Shanghai, China) with Bioflight^TM^ Western Chemiluminescent HRP Substrate (Bioworld Technology, United States).

### Immunohistochemistry

The right ovaries were dehydrated and embedded in paraffin by manual manipulation, and were sectioned at a thickness of 5 mm. The sections were deparaffinized in xylene and rehydrated through a series of graded alcohol. Endogenous peroxidase activity of the tissues was blocked with 3% H_2_O_2_ solution; 1% BSA in PBS was used to block the non-specific binding sites and subsequently incubated with appropriate primary antibodies overnight at 4°C. The ovaries were then washed with PBST and treated with secondary antibody. The protein expression was visualized with DAB staining and nucleus was stained with hematoxylin. The images were digitized by microscopy (IX73, Olympus Corporation, Shinjuku, Tokyo, Japan).

### Reagents and Antibodies

Animal PMSG and hCG were obtained from Ningbo N0.2 Hormone Factory (#110254564 and #110251282). Recombinant Human CD200 Fc Chimera Protein was purchased from R&D Systems (#2724-CD). Cellular hCG (2000 IU, H44020673) was obtained from LIVZON Pharmaceutical Group Inc. The antibodies anti-IL1 beta (ab9722), anti-Cyp19a1 (ab18995), anti-CD200 (ab203887), and anti-cyclooxygenase-2 (COX-2) (ab15191) were obtained from Abcam Tech (United Kingdom); anti-Occludin (13409-1-AP), anti-ZO-1 (21773-1-AP), anti-NF-κB p65 (10745-1-AP), and anti-VEGF (19003-1-AP) were purchased from Proteintech Tech (Wuhan, China); anti-Claudin 5 (AF5216), anti-phospho-NF-κB p65 (Ser536) (AF2006), and anti-VEGF Receptor 2 (AF6281) were obtained from Affinity Tech (United States); anto-CD200R (sc-53102), anti-Angiopoietin 1/2 (sc-271841, sc-393747), and anti-Tie2 (sc-293414) were purchased from Santa Cruz Tech (United States).

### Statistical Analysis

Data were expressed as means ± standard deviation (SD) from at least three experiments by including three or more animals per group. The figures below showed representative results. The data were analyzed using the unpaired two-tailed Student’s *t*-test. The correlation between CD200 and the number of oocytes retrieved was analyzed by Pearson’s correlation analysis. *P*-values of <0.05 were considered to be statistically significant. SPSS statistical program (version 22) was used to analyze the data.

## Results

### Proteomic Analysis Identified CD200 as Key Protein Expressed Differently in Ovaries of OHSS Model Rats After EA Treatment

**FIGURE 1:**
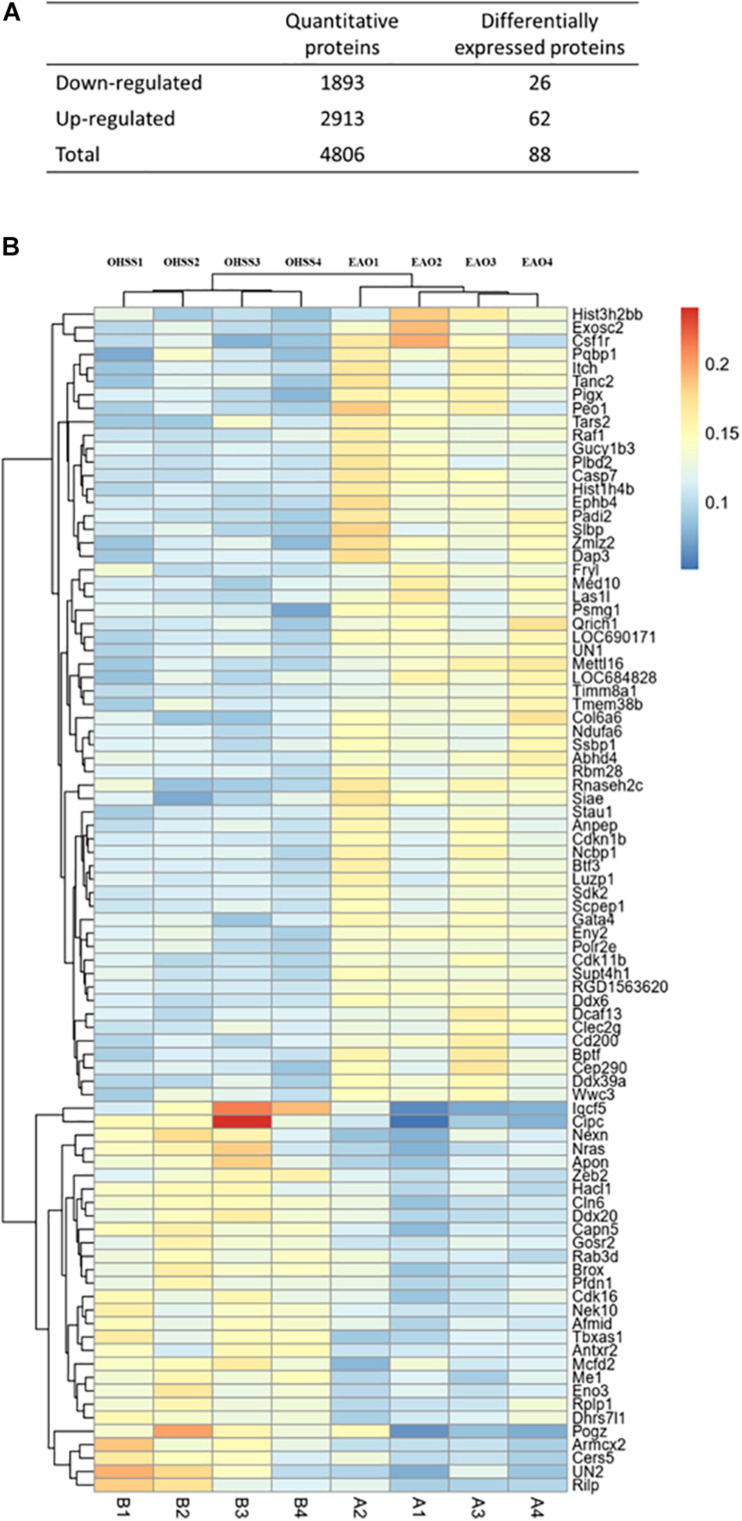
The proteins identified in ovaries of OHSS group and EAO group. **(A)** The number of proteins that were identified as differentially expressed proteins in EAO ovaries relative to OHSS group. **(B)** Hierarchical clustering presents significantly altered protein profiles identified in rat ovary, and has been analyzed by feature selection. Up-regulated and down-regulated proteins are indicated in shades of yellow and blue, respectively.

ITRAQ-labeled proteomics approach was applied in the current study to explore the key working molecules of EA treatment. A total of 4806 proteins were co-expressed in OHSS- and EA-treated ovaries, among which 88 proteins showed statistical difference (fold change >1.2) (Figure 1A). The differentially expressed proteins (DEPs) are listed in Supplementary Table 1 and were visualized using heat maps (Figure 1B). After unsupervised clustering, the EAO group showed a distinct gene expression pattern when compared with the OHSS group, suggesting a significant impact of EA treatment on protein expression.

**FIGURE 2:**
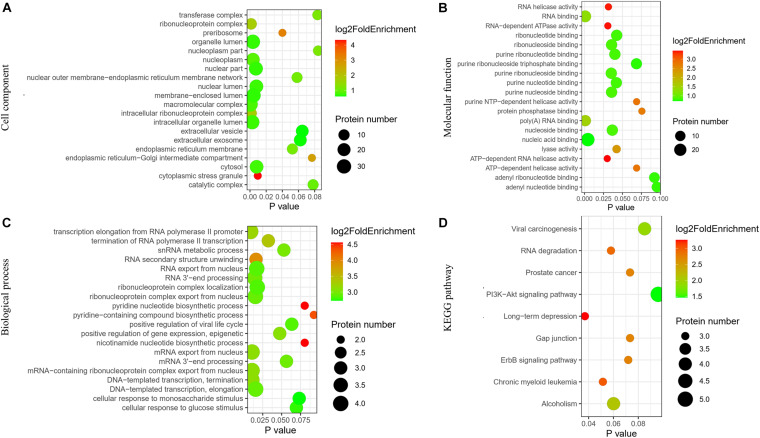
Network of identified differentially expressed proteins in ovaries of OHSS group and EAO group. **(A)** Cellular component. **(B)** Molecular functions. **(C)** Biological processes. **(D)** KEGG pathway analysis.

The pathway and GO analyses were carried out to determine the potential roles of the identified proteins. These results were shown as negative logarithm of significance, which was regarded as a statistical score and a measure of the likelihood of the genes in a given network that were found together as a result of chance, as determined by Fisher’s exact test, and those with the highest statistical significance were plotted as a bubble chart. The most common cellular component, molecular functions, and biological processes are listed in Figures 2A–C. The KEGG pathways are shown in Figure 2D.

**FIGURE 3:**
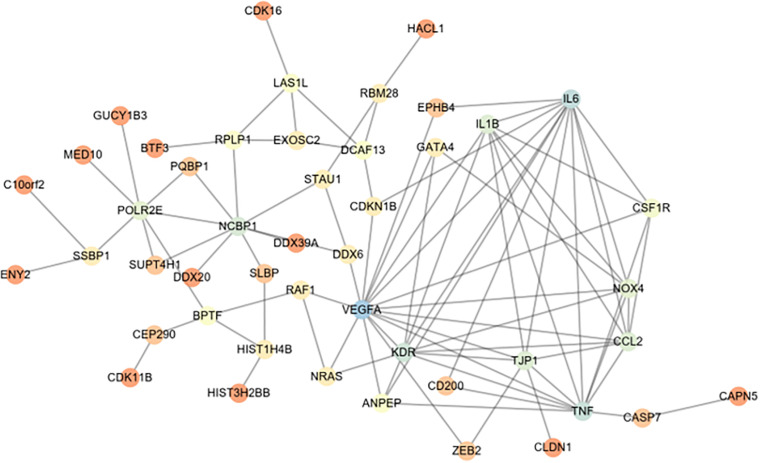
String network analysis between differentially expressed proteins (DEPs) and vasoactive substance (VEGF, IL-1β, TNF-α, etc.). The relationship between vasoactive substance including VEGF, VEGFR2, IL-6, IL-1β, TNFα, and DEPs in ovaries from EAO and OHSS model rats was analyzed by String online software.

CD200 was the key protein identified by iTRAQ analysis, and showed significant up-regulation in the ovary of OHSS rat model following EA treatment. CD200 is a member of the immunoglobulin super-family, and suppresses the immune function via its receptor CD200R (Hernangómez et al., 2014; Mousavinezhad-Moghaddam et al., 2016). The relationship between CD200 and vasoactive substance including VEGF, VEGFR, IL-6, IL-1β, and TNFα was analyzed by String online software. As shown in Figure 3, CD200 was related with TNFα and IL-6, which indicated that CD200 may be effector of EA treatment on OHSS rats.

### Decreasing CD200 Level in Follicular Fluid Obtained From IVF Patients

**FIGURE 4:**
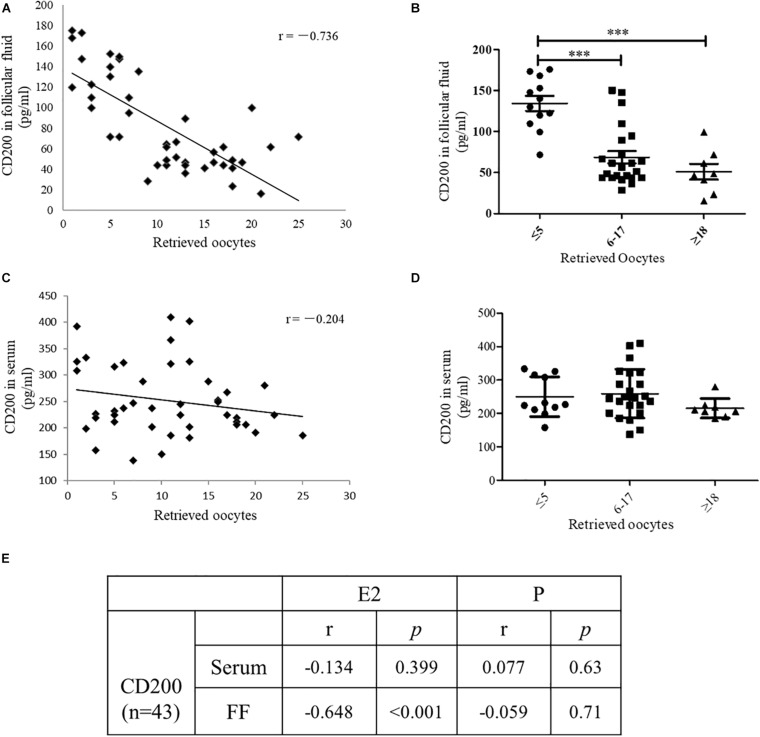
The level of CD200 in follicular fluid and serum of patients who underwent IVF procedures. Correlations between CD200 concentration and the number of oocytes retrieved in FF **(A)** and serum **(C)** (*n* = 43). The levels of CD200 in follicular fluid **(B)** and serum **(D)** were compared between the groups with higher (≥18, *n* = 12), middle (6–17, *n* = 23), and lower (≤5, *n* = 8) oocytes retrieved. The correlations between CD200 concentration and the level of E2 and P were analyzed **(E)**. ^∗∗∗^*P* < 0.001. Abbreviations: E2, estradiol; P, progesterone.

To identify whether CD200 might be involved in the development of oocyte, the level of CD200 was quantitatively determined in follicular fluid and serum of IVF patients. A total of 43 women were recruited and grouped according to the number of oocytes retrieved. The number of oocytes retrieved was categorized as ≤5 (27.9%), 6–17 (53.5%), and ≥18 (18.6%). As shown in Figure 4A, the CD200 level showed negative correlation with the number of oocytes retrieved (*r* = −0.736, *P* < 0.001). Women who produced ≤5 oocytes had a mean CD200 level of 131.7 pg/ml, which was significantly increased by 61.3% over CD200 levels in women who produced ≥18 oocytes (Figure 4B, *P* < 0.001). However, there was no significant difference in the levels of CD200 in the serum (Figures 4C,D). Similarly, Estradiol (E2), which is the risk indicator of OHSS, was negatively correlated with the levels of CD200 in follicular fluid, no correlation between progesterone (P) and CD200 (Figure 4E). These results indicated that CD200 mediates the development of OHSS; however, the internal mechanisms of CD200 in OHSS require further experiments.

### Expression of CD200/CD200R and Inflammatory Pathway Molecules in hCG-Induced KGN Cells, and Vascular Barrier-Related Proteins in HUVECs

**FIGURE 5:**
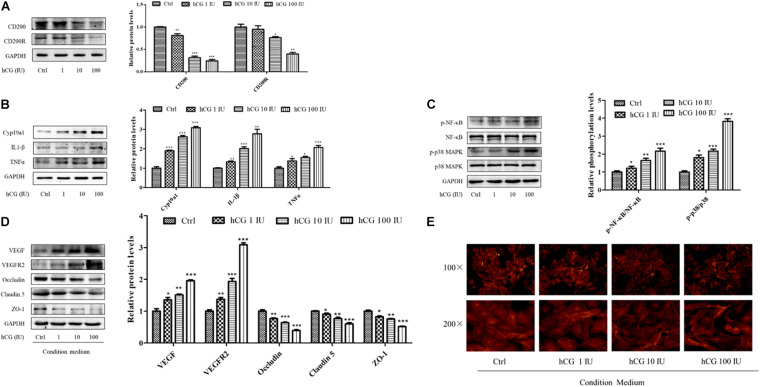
Effects of hCG on CD200 and inflammatory response pathway in KGN cells and the effects of CM on vascular permeability in HUVECs. The cells were treated with vehicle control (Ctrl) and 1, 10, and 100 IU/mL hCG for 24 h. The protein levels of CD200 and CD200R **(A)**, Cyp19a1, IL-1β, and TNFα **(B)**, and of NF-κB, p-NF-κB, p38 MAPK, and p-p38 MAPK **(C)** were examined. HUVECs were treated with condition culture medium from KGN for 24 h. **(D)** The levels of vasoactive proteins VEGF and VEGFR2, and cell junction proteins of Occludin, Claudin 5, and ZO-1 in HUVECs. **(E)** Fluorescent photomicrographs of HUVEC monolayer stained with TRITC-phalloidin with a final concentration of 20 mg/ml. Data were presented as means ± SD, *P < 0.5, **P < 0.01, ***P < 0.001 versus Ctrl.

The KGN cell line is a steroidogenic human ovarian granulose-like tumor cell line that is responsive to follicle-stimulating hormone (FSH) and secreted estradiol. HCG is the key hormone to trigger oocyte maturation. In this study, KGN cell model was used to examine the effects of hCG on CD200, its receptor, and inflammatory cytokines. KGN cells were treated with 1, 10, and 100 IU/ml hCG for 24 h, respectively. As shown in Figure 5A, the expression of CD200 and CD200R was decreased in a concentration-dependent manner. Cyp19a1 acts as a key enzyme for estradiol synthesis, and was up-regulated by hCG, which was parallel to the E2 levels in IVF patients. The protein levels of inflammatory cytokines IL-1β and TNFα showed significant up-regulation by hCG (Figure 5B). p38 MAPK and NF-κB pathway participated in regulating the inflammatory response in EA-treated OHSS rat model, and were verified in hCG-induced KGN cells. As shown in Figure 5C, hCG increased the phosphorylation levels of NF-κB and p38 MAPK. Therefore, both *in vitro* and *in vivo* study indicated an active role of hCG in stimulating inflammatory response.

The interactions between the granulosa cells and endothelial cells in the ovary were investigated via treating HUVECs with the conditional medium from culturing KGN cells under different circumstances. The medium from culturing KGN cells at concentrations of 0, 1, 10, and 100 IU/ml hCG was collected, and then HUVECs were cultured for another 24 h. The expression and protein levels of vasoactive substances VEGF and VEGFR2 and cell–cell junctional molecules Claudin 5, Occludin, and ZO-1 were determined. The culture medium with higher concentration of hCG showed increased expression of VEGF and VEGFR2, and significantly decreased protein levels of Occludin, Claudin 5, and ZO-1 (Figure 5D).

What is more, the relative distribution of HUVECs cytoskeletal f-actin that could be responsible for augmentation of vascular permeability was monitored by TRITC-phalloidin. Fluorescent photomicrographs of control group showed the existence of normal f-actin filaments, which traversed the cells and appeared to form a contiguous network of f-actin throughout the monolayer. HCG caused considerable rearrangement of f-actin in HUVECs in a concentration-dependent manner, and formed clusters in the peripheral regions of the cells (Figure 5E). The abnormal expressions of protein and the altered cytoskeletal distribution in the HUVECs created gaps between adjacent endothelial cells, which might be due to the increased secretion of inflammatory cytokines in KGN culture medium.

### CD200Fc Reduced Inflammatory Response in hCG-Induced KGN Cells and Maintained the Vascular Barrier Function in HUVECs

**FIGURE 6:**
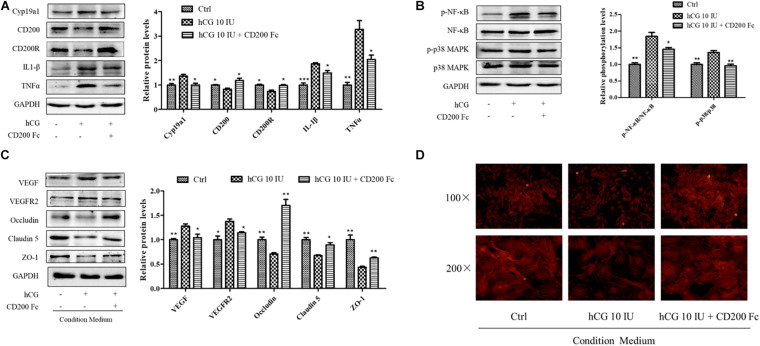
Effects of CD200Fc on inflammatory response pathway in KGN cells and vascular permeability-related protein and cell cytoskeleton in HUVECs. **(A)** The cells were treated with vehicle (Ctrl), and 10 IU/ml hCG with or without 100 ng/ml CD200Fc for 24 h, and the protein levels of CD200, CD200R, Cyp19a1, IL-1β, and TNFα were examined by western blotting. **(B)** The ovarian expression of NF-κB, p-NF-κB, p38 MAPK, and p-p38 MAPK, and the relative NF-κB and p38 MAPK activity were derived as p-NF-κB and p-p38 MAPK, and are expressed as fold changes over Ctrl. HUVECs were treated with condition culture medium from KGN (10 IU/ml hCG with or without 100 ng/ml CD200Fc) for 24 h. **(C)** The levels of vasoactive proteins VEGF and VEGFR2, and cell junction proteins Occludin, Claudin 5, and ZO-1 were examined by western blotting. **(D)** Fluorescent photomicrographs of HUVECs monolayer stained with TRITC-phalloidin with a final concentration of 20 mg/ml. Data were presented as means ± SD, *P < 0.5, **P < 0.01, ***P < 0.001 versus Ctrl.

CD200Fc, a CD200R agonist, was used to investigate the anti-inflammatory effect in KGN cells with a concentration of 100 ng/ml. As shown in Figure 6A, stimulation with hCG significantly decreased both CD200R and CD200 protein levels. Pre-treatment with CD200Fc for 1 h before addition of 10 IU/ml hCG showed significant up-regulation of CD200R protein expression, and reduction of inflammatory mediators IL-1β and TNFα levels simultaneously. The increased expression of Cyp19a1 stimulated by hCG was inhibited by CD200Fc as well. The inflammatory response pathway NF-κB and p38 MAPK activities were determined, and the phosphorylation of NF-κB and p38 MAPK was up-regulated by hCG but significantly down-regulated with treatment of CD200Fc (Figure 6B). Therefore, CD200Fc was considered to play an anti-inflammatory role in hCG-induced KGN cells by promoting CD200-CD200R interaction and inhibition of p38 MAPK/NF-κB signaling pathway-mediated cytokines expression.

To investigate the effects of CD200Fc on the interaction between granulosa cells and vascular endothelial cells, CM from CD200Fc-treated KGN cells was collected for culturing the HUVECs. In HUVECs, vasoactive substances VEGF and VEGFR2 expression were decreased when cultured with CM from CD200Fc-treated cells, and the cell junctional proteins Occludin, Claudin 5, and ZO-1 were significantly increased (Figure 6C). Fluorescent photomicrographs of HUVEC monolayer with TRITC-phalloidin showed that hCG treatment caused considerable rearrangement of f-actin, and CM from CD200Fc treated cells obviously amended the abnormal cytoskeletal distribution in the HUVECs (Figure 6D). Therefore, the improvement in vascular barrier function of HUVECs might attribute to the amended cell cytoskeletons and junctional proteins, especially the inhibition of inflammatory expression in KGN cell.

### EA Decreased the Number of Corpus Luteal and Increased the Expression of CD200 and CD200R in Ovary

**FIGURE 7:**
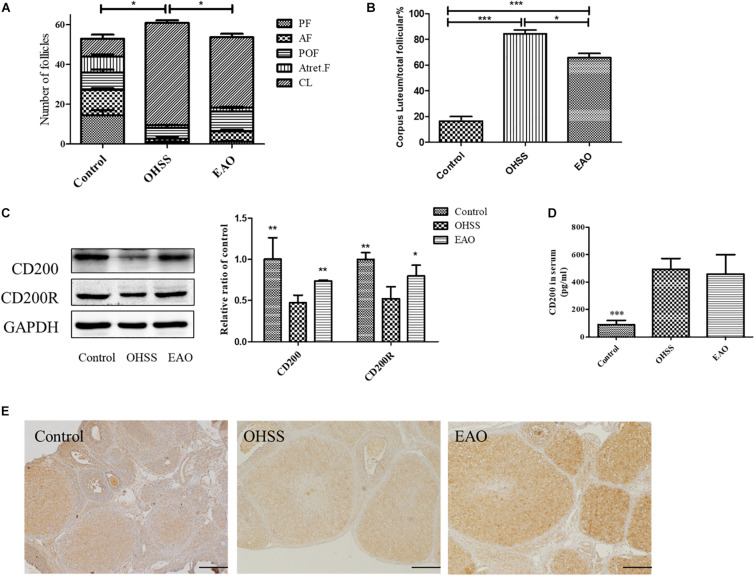
The number of follicles at different stages, expression of CD200 and CD200R in EA-treated OHSS model. **(A)** The number of follicles at different stages in each group. **(B)** The ratio of corpora lutea/total follicles. **(C)** The protein expression of CD200 and CD200R in ovary of OHSS rats with or without EA treatment. **(D)** The CD200 levels in the serum of experimental groups were detected by ELISA. **(E)** The distribution of CD200 was detected by IHC, original magnification 40×, scale bar = 100 μm. Data were shown as means ± SD, *P < 0.5, **P < 0.01, ***P < 0.001 versus OHSS group.

The number of CL and follicles at different stages including preantral follicle (PF), antral (AF), preovulatory follicle (POF), and atretic follicle (Atret. F) was counted. There were no differences among the number of follicles at different stages of development among groups, although the number of CL and total follicles in OHSS group was significantly higher than control and EAO group (Figure 7A). EA treatment significantly decreased the number of CL, which was higher in OHSS group (*P* < 0.05, Figure 7B). The expression of CD200 in the experimental groups was further confirmed by western blotting. Ovarian expression of CD200 and CD200R was significantly decreased in OHSS group, while EA treatment increased its levels (Figure 7C). Similarly, the results of western blotting and IHC were in agreement with those of iTRAQ (Figure 7E). However, no significance was observed in the CD200 levels in the serum between OHSS and EAO groups (Figure 7D). Those results suggest that CD200-CD200R axis might participate in the regulation of ovulation and luteinization.

### EA Decreased the Inflammatory Response and Improved the Expression of Vascular Permeability-Related Proteins in Ovary

**FIGURE 8:**
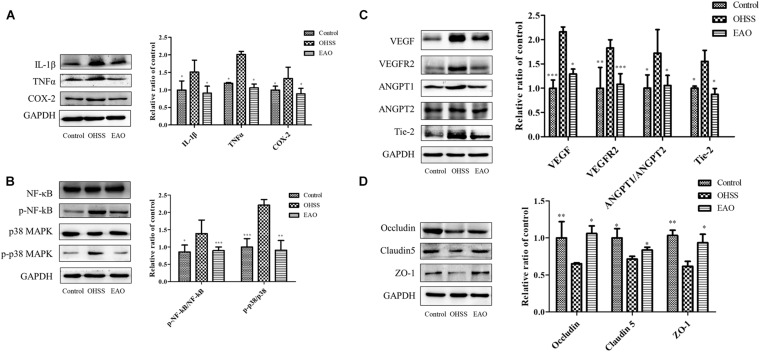
Effects of EA treatment on the expression of inflammatory pathway and cell junction proteins in ovary of OHSS rats. **(A)** The expressions of IL-1β, TNFα, and COX-2 protein in ovary were determined by western blotting. **(B)** The ovarian expression of NF-κB, p-NF-κB, p38 MAPK, and p-p38 MAPK, and the relative NF-κB and p38 MAPK activity were derived as p-NF-κB and p-p38 MAPK. The protein expression of vasoactive factor VEGF, VEGFR2, ANGPT1, ANGPT2, and Tie-2 **(C)** and junctional protein Occludin, Claudin 5, and ZO-1 **(D)** were determined. The data were shown as means ± SD, *P < 0.5, **P < 0.01, ***P < 0.001 versus OHSS group.

Inflammatory response in the ovaries of OHSS rat model was investigated. Inflammatory cytokines, including IL-1β, TNFα, and COX-2, were determined by western blotting. The results showed significantly increased levels of IL-1β (*P* < 0.05), TNFα (*P* < 0.05), and COX-2 (*P* < 0.05) in OHSS groups when compared with control group. Furthermore, EA treatment reduced the expressions of cytokines as described above (Figure 8A). NF-κB has been an important modulator in the regulation of inflammatory response (Hayden and Ghosh, 2008). NF-κB activation was detected to further explore the anti-inflammatory mechanism of EA treatment in the ovary of OHSS rats (Figure 8B). The results showed that ovarian hyperstimulation markedly induced the phosphorylation of NF-κB, and the effect was significantly reserved by EA treatment. On the other hand, the phosphorylation of p38 MAPK in OHSS ovaries by significantly attenuating with EA treatment. The results described above indicated that EA played a strong anti-inflammatory response role in OHSS rats and was mediated by p38 MAPK/NF-κB pathway.

Treatment with EA resulted in statistically significant decrease in VEGF and VEGFR2 (*P* < 0.05 and *P* < 0.01, respectively). The ANGPTs play an important role in the angiogenesis and blood vessel stabilization during luteal development. In the OHSS group, the ratio of ANGPT-1/ANGPT-2 and the expression of Tie 2 were significantly increased, while EA treatment significantly decreased this ratio and Tie 2 level (*P* < 0.05) (Figure 8C). EA treatment significantly prevented the decrease of Occludin, Claudin-5, and ZO-1 in ovaries (Figure 8D). Therefore, EA treatment improved the vascular hyper-permeability of hyperstimulated ovary mainly by reducing the expressions of vasoactive substances and increasing the levels of junctional proteins.

## Discussion

In order to comprehensively explain the role and mechanism of EA treatment, ovarian proteomic analysis of OHSS rat model with EA was performed. CD200 was significantly down-regulated in the ovaries of OHSS rat model, while up-regulated by EA treatment, and CD200 was identified as potential effector in ovary. CD200 is a membrane glycoprotein of the immune-globulin superfamily with immune suppression effect via its receptor CD200R. It has been shown that soluble CD200 fusion protein (CD200Fc), which is a CD200R agonist, could activate CD200R expression and inhibits the release of the pro-inflammatory cytokines in kinds of cells, including vascular endothelia cells (Ding et al., 2015; Jiang et al., 2016; Xu et al., 2016). In human cumulus–oocyte complex, CD200 mainly expressed in granulosa cells (Assou et al., 2006), same as our result that CD200 highly expressed in luteal granulosa cells of ovaries from EA-treated model rats. To clarify the specific role of CD200, we first detected the level of CD200 in follicular fluid, and found negative correlation with oocyte retrieved and serum E2, which are the risk factors of OHSS. These key findings demonstrated that follicular CD200 has protective effects during OHSS development, but no relevant reports have been present till date. Therefore, it is interesting to evaluate the specific role of CD200 in oocyte development in further experiments, and it may serve as a prognostic predictor for OHSS.

It has been evidenced that OHSS patients and rat models have high levels of inflammatory cytokines in the serum and FF (Artini et al., 2002), and hCG directly induces hyper-ovulation resulted in inflammation-like reaction (Orvieto, 2004). We showed that CD200 was related with inflammatory cytokines TNFα and IL1β by string analysis. *In vitro*, the expression level of inflammatory cytokines and related pathway were confirmed in KGN cells and could induce in response to the administration of hCG in a concentration-dependent manner. What is more, OHSS rat model showed decreased ovarian expression of CD200 and CD200R, and accompanied by increased inflammatory response and activated NF-κB pathway. Hence, CD200-CD200R-mediated anti-inflammatory response contributes to the reduction of cytokines in the ovary.

Inflammation triggers a wide range of physiological and pathological processes. Ovulation represents a short-term inflammatory event limited by endogenous mediators that facilitate resolution of inflammation. It is triggered in by the preovulatory surge of LH and culminates in the release of a mature fertilizable oocyte and formation of the corpus luteum. COX2 is one of the ovulation-related genes that produce prostaglandin E2 (PGE2). In this study, we demonstrated that the number of follicles at different stages and CL were significantly reduced in EAO model rats. COX-2 is stimulated in granulosa cells by hCG, and the ovarian expression was decreased following EA treatment. In the previous study, we have indicated that EA treatment decreased the levels of PGE2 in serum and ovary. From the above results, we speculated that EA treatment inhibited the oocyte ovulation then reduced the CL formation in OHSS model rat might through via anti-inflammatory responses in ovary.

In ovary, the developing CL incorporates a tightly regulated system of cellular communication between granulosa cells and endothelial cells. During ovulation, vascular growth factor families are necessary paracrine mediators, and vascular changes are a critical component of inflammation as well (Duffy et al., 2019). In OHSS, hyper-permeability results in ovarian and peripheral vascular leakage, and endothelial cell barrier dysfunction was mainly responsible for these abnormalities (Albert et al., 2002). Inflammatory cytokines and vasoactive proteins VEGF and ANGPTs play crucial roles in OHSS (Lebovic et al., 1999; Gomez et al., 2002). Vascular endothelial cells act as the target response to inflammatory mediators and vasoactive substances, leading to vascular leakage (Albert et al., 2002; Gomez et al., 2002; Kumar et al., 2009). Our data pointed out that increased expression of inflammatory pathway and vasoactive proteins was all restored in model rats with EA treatment. Pre-treatment with CD200Fc significantly reduced the VEGF/VEGFR2 levels and up-regulated cell junction proteins, and furthermore restored the cytoskeleton arrangement in HUVECs. Considering the results of inhibition of inflammation in KGN cells, it can be considered that the maintenance of vascular barrier function in HUVECs was partially attributed to the attenuated inflammatory response in granulosa cells by CD200Fc. Accordingly, the ovarian and granulosa cells as the source of inflammatory cytokines, CD200 protected VEB function via anti-inflammatory effect.

## Conclusion

Collectively, we propose for the first time that CD200 is one of the key molecules by which EA exerts clinical efficacy and elucidates the effector mechanisms by which CD200 regulates granulosa cell inflammatory responses that in turn affect ovulation and vascular barrier function. This provides a scientific basis for CD200 as an effector target in EA treatment.

## Data Availability Statement

The mass spectrometry proteomics data have been deposited to the ProteomeXchange Consortium (http://proteomecentral.proteomexchange.org) via the iProX partner repository with the dataset identifier PXD023493 (http://proteomecentral.proteomexchange.org/cgi/GetDataset?ID=PXD023493).

## Ethics Statement

All animal studies (including the rat euthanasia procedure) were done in compliance with the regulations and guidelines of Nanjing Jinling Hospital and were conducted according to the AAALAC and the IACUC guidelines (reference number 2019JLHGKJDWLS-005). Samples were collected after obtaining the written informed consent from the patients and ethics board approval from the Nanjing Jinling Hospital.

## Author Contributions

LC and BY designed the experiments. LC, XH, and LW performed the experiments, analyzed the data, and wrote the manuscript. CW, MG, XT, JJ, RM, and XG helped for the preparation of the manuscript. All authors read and approved the final version of the article.

## Conflict of Interest

The authors declare that the research was conducted in the absence of any commercial or financial relationships that could be construed as a potential conflict of interest.

## Footnotes

1 http://www.matrixscience.com

2 http://proteomecentral.proteomexchange.org

3 http://proteomecentral.proteomexchange.org/cgi/GetDataset?ID=PXD023493
